# Pulmonary response to intratracheal instillation of ultrafine versus fine titanium dioxide: role of particle surface area

**DOI:** 10.1186/1743-8977-5-17

**Published:** 2008-12-01

**Authors:** Tina M Sager, C Kommineni, Vincent Castranova

**Affiliations:** 1 Health Effects Laboratory Division, National Institute for Occupational Safety and Health, Morgantown, West Virginia, USA; 2 Department of Environmental Health, Harvard School of Public Health, Boston, Massachusetts, USA

## Abstract

**Background:**

The production and use of nanoparticles is growing rapidly due to the unique physical and chemical properties associated with their nano size and large surface area. Since nanoparticles have unique physicochemical properties, their bioactivity upon exposure to workers or consumers is of interest. In this study, the issue of what dose metric (mass dose versus surface area dose) is appropriate for toxicological studies has been addressed. Rats were exposed by intratracheal instillation to various doses of ultrafine or fine TiO_2_. At 1, 7, or 42 days post-exposure, inflammatory and cytotoxic potential of each particle type was compared on both a mass dosage (mg/rat) as well as an equal surface area dosage (cm^2 ^of particles per cm^2 ^of alveolar epithelium) basis.

**Results:**

The findings of the study show that on a mass basis the ultrafine particles caused significantly more inflammation and were significantly more cytotoxic than the fine sized particles. However, when doses were equalized based on surface area of particles delivered, the ultrafine particles were only slightly more inflammogenic and cytotoxic when compared to the fine sized particles. Lung burden data indicate that ultrafine TiO_2 _appears to migrate to the interstitium to a much greater extent than fine TiO_2_.

**Conclusion:**

This study suggests that surface area of particles may be a more appropriate dose metric for pulmonary toxicity studies than mass of particles.

## Background

Nanoscience and nanotechnology offer new opportunities for making superior materials for use in industrial and health applications [1,2]. As the field of nanotechnology vastly expands, many questions involving the effects of nanomaterials on the environment as well as human health have been raised which warrant investigation. The primary problem plaguing the field of nanotechnology is the possibility that nanoparticles can become suspended in air during production, incorporation into consumer products, and use or disposal of such products. Therefore, manufactured nanoparticles can become a component of the indoor and outdoor environments and, thus, the air we breathe [2].

A number of toxicology studies have suggested that for some classes of materials, biological response following deposition in the lungs is dependent on particle surface area [3,4]. Materials of interest typically possess characteristics such as low solubility, which leads to extended persistence in the lungs. These materials can be produced as ultrafine particles, which have high specific surface areas [5]. Titanium dioxide (TiO_2_) is an example of a fine, low solubility particle which was considered to exhibit relatively low toxicity [6]. However, in a key study, Ferin et al. [7] demonstrated that ultrafine TiO_2 _(UFTiO_2_) caused more inflammation in rat lungs than exposure to the same airborne mass concentration of fine TiO_2 _(FTiO_2_). Up until this point in time, TiO_2 _often had been used as a low solubility, low toxicity, control dust in many studies. Therefore, this report was highly influential in highlighting that a material that was low in solubility and toxicity in the form of fine particles could be bioactive in the form of ultrafine particles [8].

In the last decade, it has become clear from rat studies that chronic exposure to high airborne mass concentrations of low solubility, low toxicity particles, such as carbon black and titanium dioxide, can lead to development of the features characteristic of "rat lung overload" [9]. A breakdown in normal alveolar macrophage- mediated clearance is seen in overload. This particle-induced depression of clearance was proposed to be a consequence of volumetric overload of the alveolar macrophages and the resulting loss of alveolar macrophage mobility [10]. This overload phenomenon was thought to be a problem related to particles occupying a large volume inside each macrophage and, thus, preventing them from phagocytosing additional particles and moving to the mucociliary escalator to be cleared. This was known as the volumetric overload hypothesis [8,10]. However, more recent studies suggest that volumetric overload is not a complete explanation for the toxicological response to ultrafine particles. Recent evidence has suggested that surface area may drive inflammation [11]. Exposure of rats to nanoparticles, with a large surface area per mass, showed features of poor clearance and inflammation at a lower lung burden, in terms of mass and volume of particles, compared with larger size particles with the same mineral and chemical properties [4].

A study conducted by Tran et al. [11] compared the inflammatory response to inhalation of two sizes of two very different types of low solubility, low toxicity particles. At an equal lung mass burden, the smaller particles were more inflammatory than the larger particles. However, when the lung burden was expressed as total particle surface area delivered, the inflammatory potential of the large and small particles was similar [11]. This supports the hypothesis that surface area of the particle may be an important component of toxic potential. In contrast, *in vitro* studies by Monteiller et al. [9] utilized insoluble quartz, which has a very high surface reactivity. Due to this very high surface reactivity, quartz stimulated release of inflammatory mediators at a much lower mass and surface area dose than did low solubility, low toxicity particles. This is consistent with the high inflammatory potential of quartz in animal models. The outcome of this study indicates that, while surface area is important, surface activity is also a critical factor to assess pulmonary toxicity of particles. Studies supporting an important role of particle surface area in the bioactivity of low solubility, low toxicity particles, such as ultrafine carbon black and ultrafine titanium dioxide, impact assessment of workplace and environmental exposure to these nanoparticles, since regulations are currently based on mass of airborne particles [9].

In summary, several studies have concluded that on an equal mass dose basis ultrafine or nano-sized particles are uniquely more toxic than fine-sized particles of the same composition due to a high particle surface area for the nanoparticles [4,7-9,11]. Recently, this conclusion has been questioned [12,13]. It is possible that these conflicting results may be due to the use of highly agglomerated UFTiO_2 _samples in the later two studies [12,13]. Therefore, the aim of the present study was to readdress the issue of the relative toxicity of UFTiO_2 _vs FTiO_2 _on a mass dose or equivalent particle surface area delivered dose basis using alveolar lining fluid, which our laboratory has shown previously greatly improves nanoparticles dispersion [14], as the particle suspension medium. In the present study, *in vivo* intratracheal exposures of rats to ultrafine and fine titanium dioxide were conducted. Animals were given a dose of particles based on mass, and dose was normalized to surface area of particles administered. Particle-induced changes in several pulmonary toxicity parameters were compared on a mass dose and surface area of particles administered basis to determine which dose metric is more appropriate in nanoparticle toxicology studies.

## Results

**Figure 1:**
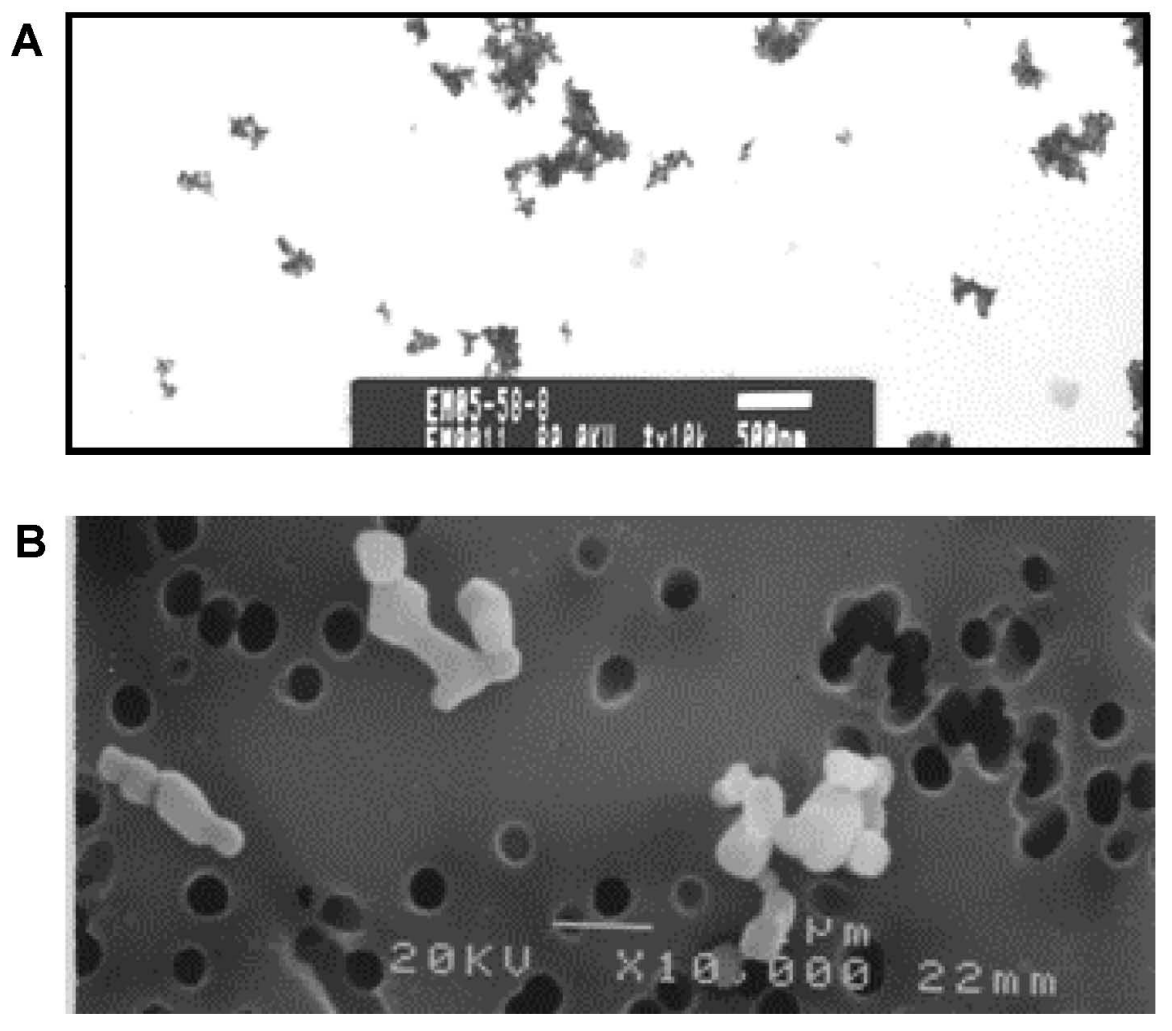
**Electron micrographs of titanium dioxide particles suspended in BALF**. A transmission electron micrograph of UFTiO_2 _suspended in BALF (A). Magnification of 60,000 × (note 500 nm scale bar). A scanning electron micrograph of FTiO_2 _suspended in BALF (B). Magnification of 20,000 × (note 1 um scale bar).

UFTiO_2 _and FTiO_2 _suspended in acellular BALF were administered to Fischer 344 rats via intratracheal instillation to assess pulmonary toxicity. BALF was used as the suspension medium, because it greatly improves dispersion of TiO_2 _[14]. Figure 1 shows electron micrographs of the ultrafine (1A) and fine (1B) particles in suspension, indicating that the majority of ultrafine particles agglomerates were in the 200–300 nm range while the fine particles exceeded 1 um in diameter. The dose of particles administered was expressed on a mass basis (mg/rat) and was also normalized to surface area of particles administered per alveolar epithelial surface area (cm^2^/cm^2^). The surface areas of the respective particles were determined by the BET gas absorption technique with values of 48.08 m^2^/g for UFTiO_2 _and 2.34 m^2^/g for FTiO_2 _[15]. This comparison of mass and surface area doses was conducted to assess whether surface area of particles administered is the more accurate dose metric that should be considered when assessing nanoparticle pulmonary toxicity parameters. Pulmonary response parameters measured included, indicators of pulmonary inflammation (PMN number or inflammatory mediators, such as TNF-α, MIP-2, and IL-1β), markers of lung injury (LDH activity or albumin levels) and markers of macrophage activity (zymosan-stimulated chemiluminescence and NO-dependent chemiluminescence).

### Comparison of pulmonary toxicity of UFTiO_2 _and FTiO_2_

**Figure 2:**
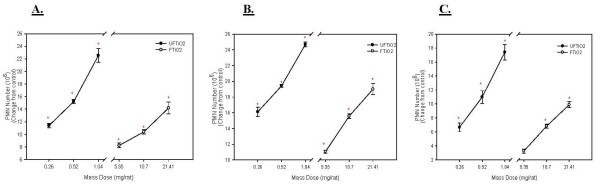
**A comparison of inflammation elicited in animals receiving various mass doses of UFTiO_2 _and FTiO_2 _suspended in BALF**. A comparison of inflammation elicited in animals receiving various mass doses of UFTiO_2 _and FTiO_2 _suspended in BALF at 1 day (Panel A), 7 days (Panel B), and 42 days (Panel C) post-exposure. Rats were exposed to various mass doses of UFTiO_2 _and FTiO_2 _by intratracheal instillation. Animals were euthanized at 1 day, 7 days, and 42 days post-exposure and bronchoalveolar lavage was performed. Inflammation was assessed by BAL PMN counts. Values are increased PMN number above the BALF control and are given as means ± SE of 8 experiments. Control PMN values were 1.37 ± 0.098 × 10^6^, 0.78 ± 0.074 × 10^6^, and 0.88 ± 0.095 × 10^6 ^cells/rat for 1, 7 and 42 days, respectively. Linear regression analysis with a 95% confidence interval reveals that on a mass dose basis UFTiO_2 _causes significantly more inflammation than FTiO_2 _at all post-exposure time points. * indicates a significant increase from control (p < 0.05; ANOVA).

**Figure 3:**
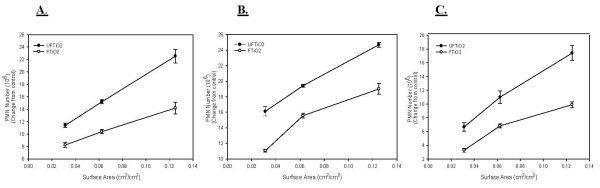
**A comparison of inflammation elicited in animals receiving doses of UFTiO_2 _and FTiO_2 _normalized to surface area of particles administered per surface area of alveolar epithelium**. A comparison of inflammation elicited in animals receiving doses (0.0313, 0.0625 and 0.125 cm^2^/cm^2^) of UFTiO_2 _and FTiO_2 _normalized to surface area of particles administered per surface area of alveolar epithelium at 1 day (Panel A), 7 days (Panel B), and 42 days (Panel C) post-exposure. Particles were suspended in BALF. Rats were exposed to various doses of UFTiO_2 _and FTiO_2 _by intratracheal instillation. Animals were euthanized at 1 day, 7 days, and 42 days post-exposure and bronchoalveolar lavage was performed. Inflammation was assessed by BAL PMN counts. Values are increased PMN number above the BALF control and are given as means ± SE of 8 experiments. Control PMN values were 1.37 ± 0.098 × 10^6^, 0.78 ± 0.074 × 10^6^, and 0.88 ± 0.095 × 10^6 ^cells/rat for 1, 7 and 42 days, respectively. Linear regression analysis with a 95% confidence interval reveals that, when dose is normalized to surface area of particles administered, dose responses curves assessing inflammation caused by UFTiO_2 _and FTiO_2 _exposure are not significantly different. On a dose normalized to surface area, UFTiO_2 _elicits at most a 2 fold increase in inflammation when compared to FTiO_2 _at all post-exposure times.

On a mass dose basis, UFTiO_2 _and FTiO_2 _both produced a dose-dependent increase in PMN harvested by BAL (Figure 2). All doses of UFTiO_2 _caused a significant increase in PMN over control at all post-exposure time periods. For FTiO_2_, the medium and high doses caused a significant increase in PMN number over control at all post-exposure days. However, it took a significantly larger mass dose of FTiO_2 _to obtain the same inflammogenic response as the UFTiO_2 _particles (Figure 2). When comparing this inflammogenic response for UFTiO_2 _exposure to FTiO_2 _exposure, on a mass dose basis (for example the PMN response to 10.7 mg/rat FTiO_2 _vs 0.26 mg/rat UFTiO_2 _from figure 2), UFTiO_2 _was shown to be 41 times more potent than the FTiO_2 _at all exposure times (Table 1). However, when dose of particles was normalized to surface area of particles administered the difference in inflammogenic responses, assessed by PMN number, of the two particle types was much less (Figure 3). Indeed, a linear regression curve analysis with a 95% confidence interval showed that there was no significant difference between the dose-response curves for UFTiO_2 _and FTiO_2 _when dose was expressed as total particle surface area delivered (Figure 3). In fact, when dose was normalized to surface area of particles administered, the inflammogenic response elicited by UFTiO_2 _was only 2-fold greater than the FTiO_2 _response at 1 day post-exposure, 1.3-fold greater at 7 days post-exposure and 1.6-fold greater at 42 days post-exposure (Table 1).

**Table 1:** Potency difference between UFTiO_2 _and FTiO_2 _when analyzed on a mass vs. surface area basis.

**Parameter**	**Dose Metric**	**1 Day**	**7 Days**	**42 Days**
**PMN (10^6^)**				
	**Mass**	41	41	41
	**Surface Area**	2	1.3	1.6
				
**LDH (U/l)**				
	**Mass**	41	30	41
	**Surface Area**	1.2	1.2	1.8
				
**Albumin**				
	**Mass**	82	41	41
	**Surface Area**	3	1.4	2
				
**TNF-α (pg/ml)**				
	**Mass**	41	41	41
	**Surface Area**	1.2	1.4	1.5
				
**MIP-2 (pg/ml)**				
	**Mass**	41	41	82
	**Surface Area**	1.2	1.1	1.1
				
**IL-1β (pg/ml)**				
	**Mass**	82	82	82
	**Surface Area**	1.6	1.8	1.4
				
**Zym. Stim. Chemi.**				
	**Mass**	82	82	82
	**Surface Area**	1.75	1.8	3
				
**NO Dep. Chemi.**				
	**Mass**	41	82	82
	**Surface Area**	1.5	7	8

Comparison of UFTiO_2 _and FTiO_2 _potency differences. The data were analyzed to show the potency difference between UFTiO_2 _and FTiO_2 _on a mass basis as well as the fold increase in pulmonary toxicity response on a surface area basis. All post-exposure time points were analyzed and are reported in the table. On a mass basis, the UFTiO_2 _has much greater potency than FTiO_2_, but when dose is normalized to total particle surface area administered the fold increase in response between the UFTiO_2 _and FTiO_2 _is greatly reduced.

**Figure 4:**
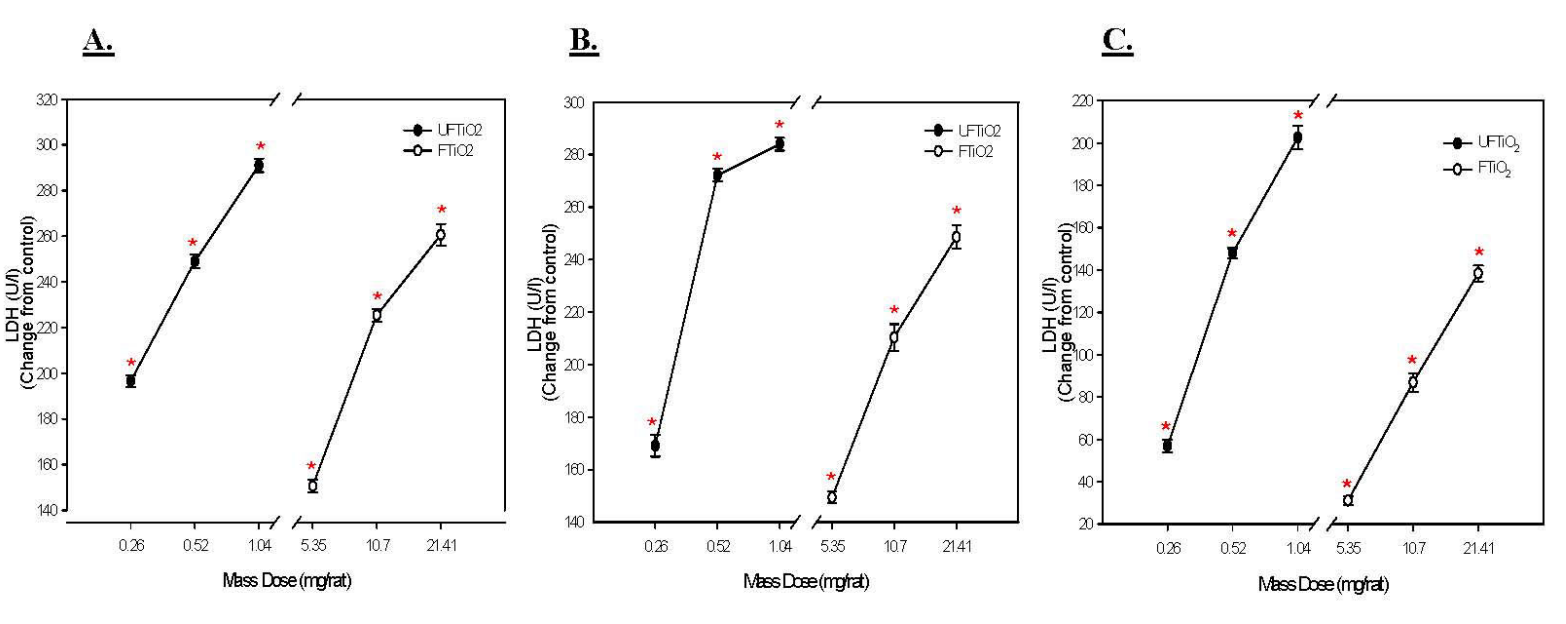
**A comparison of cellular cytotoxicity elicited in animals receiving various mass doses of UFTiO_2 _and FTiO_2 _suspended in BALF**. A comparison of cellular cytotoxicity elicited in animals receiving various mass doses of UFTiO_2 _and FTiO_2 _suspended in BALF at 1 day (Panel A), 7 days (Panel B), and 42 days (Panel C) post-exposure. Rats were exposed to various mass doses of UFTiO_2 _and FTiO_2 _by intratracheal instillation. Animals were euthanized at 1 day, 7 days, and 42 days post-exposure and bronchoalveolar lavage was performed. Cytotoxicity was assessed by measuring LDH activity. Values are increase in LDH activity above the BALF control and are given as means ± SE of 8 experiments. Control values of LDH activity were 46.375 ± 2.24, 39.5 ± 1.35, and 37.25 ± 2.63 U/l for 1, 7 and 42 days, respectively. Linear regression analysis with a 95% confidence interval reveals that on a mass dose basis UFTiO_2 _causes significantly more LDH activity than FTiO_2 _at all post-exposure time points. * indicates a significant increase from control (p < 0.05; ANOVA).

**Figure 5:**
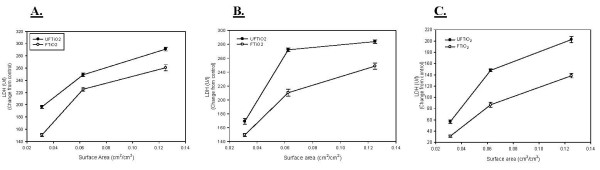
**A comparison of cytotoxicity elicited in animals receiving doses of UFTiO_2 _and FTiO_2 _normalized to surface area of particle administered per surface area of alveolar epithelium**. A comparison of cytotoxicity elicited in animals receiving doses (0.0313, 0.0625 and 0.125 cm^2^/cm^2^) of UFTiO_2 _and FTiO_2 _normalized to surface area of particles administered per surface area of alveolar epithelium at 1 day (Panel A), 7 days (Panel B), and 42 days (Panel C) post-exposure. Animals were exposed to various doses of UFTiO_2 _and FTiO_2 _by intratracheal instillation. Animals were euthanized at 1 day, 7 days, and 42 days post-exposure and bronchoalveolar lavage was performed. Cytotoxicity was assessed by measuring LDH activity. Values are increase in LDH activity above the BALF control and are given as means ± SE of 8 experiments. Control values of LDH activity were 46.375 ± 2.24, 39.5 ± 1.35, and 37.25 ± 2.63 U/l for 1, 7 and 42 days, respectively. Linear regression analysis with 95% confidence internal reveals that, when dose is normalized to surface area of particles administered, responses to UFTiO_2 _and FTiO_2 _are not significantly different.

Lactate dehydrogenase (LDH) activity was measured to assess cellular cytotoxicity after TiO_2 _exposures. As with PMN, both UFTiO_2 _and FTiO_2 _caused a dose dependent increase in LDH activity in BALF. For LDH activity, on a mass dose basis, a significantly greater mass dose of FTiO_2 _was required at all post-exposure time points to obtain the same responses as seen with UFTiO_2 _exposure (Figure 4). For LDH activity, on a mass dose basis, UFTiO_2 _exposure was shown to be approximately 30–41 times more potent at all post-exposure time points than FTiO_2 _exposure (Table 1). However, when dose of particles was normalized to surface area of particles administered the difference in LDH activity of the two particle types was much less (Figure 5). Indeed, a linear regression curve analysis with a 95% confidence interval showed that there was no significant difference between the dose-response curves for UFTiO_2 _and FTiO_2 _when dose was expressed as total particle surface area delivered (Figure 5). In fact, when dose was normalized to surface area of particles administered, the UFTiO_2 _exposure produced LDH activity levels that were at most only 1.8-fold greater than the FTiO_2 _exposure at all post-exposure time points analyzed (Table 1).

**Figure 6:**
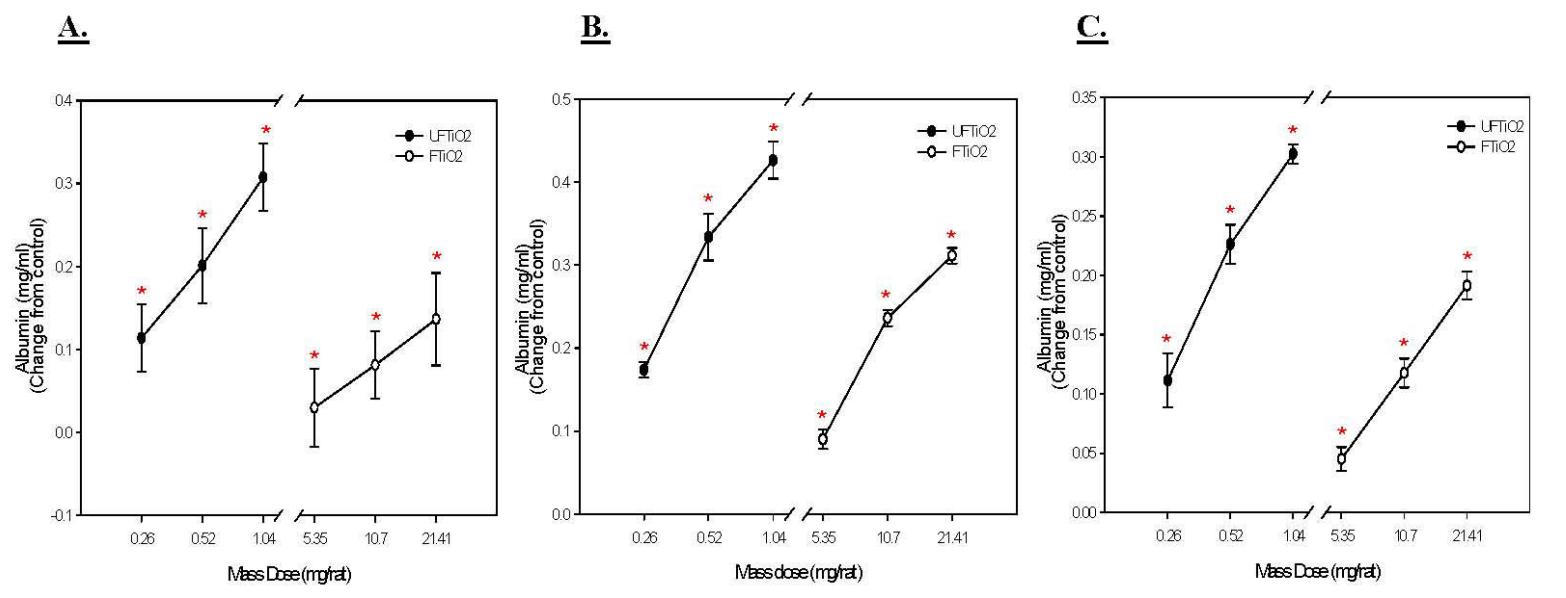
**A comparison of cellular damage elicited in animals receiving various mass doses of UFTiO_2 _and FTiO_2 _suspended in BALF**. A comparison of cellular damage elicited in animals receiving various mass doses of UFTiO2 and FTiO_2 _suspended in BALF at 1 day (Panel A), 7 days (Panel B), and 42 days (panel C) post-exposure. Rats were exposed to various mass doses of UFTiO_2 _and FTiO_2 _by intratracheal instillation. Animals were euthanized at 1 day, 7 days, and 42 days post-exposure and bronchoalveolar lavage was performed. Cellular injury was assessed by measuring albumin levels. Control values of albumin levels were 0.073 ± 0.033, 0.084 ± 0.003, 0.098 ± 0.007 mg/ml for 1, 7, and 42 days, respectively. Values are increase in albumin levels above the BALF control and are given as means ± SE of 8 experiments. * indicates a significant increase from control (p < 0.05; ANOVA).

**Figure 7:**
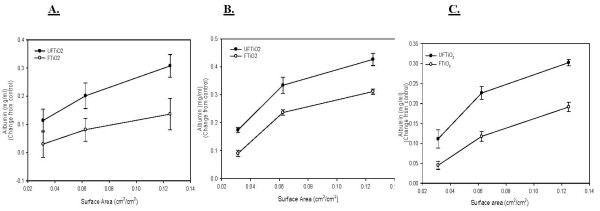
**A comparison of cellular damage elicited in animals receiving doses of UFTiO_2 _and FTiO_2 _normalized to surface area of particle administered per surface area of alveolar epithelium**. A comparison of cellular damage elicited in animals receiving doses (0.0313, 0.0625 and 0.125 cm^2^/cm^2^) of UFTiO2 and FTiO_2 _normalized to surface area of particles administered per surface area of alveolar epithelium at 1 day (Panel A), 7 days (Panel B), and 42 days (Panel C) post-exposure. Rats were exposed to various mass doses of UFTiO_2 _and FTiO_2 _by intratracheal instillation. Animals were euthanized at 1 day, 7 days, and 42 days post-exposure and bronchoalveolar lavage was performed. Cellular injury was assessed by measuring albumin levels. Control values of albumin levels were 0.073 ± 0.033, 0.084 ± 0.003, 0.098 ± 0.007 mg/ml for 1, 7, and 42 days, respectively. Values are increase in albumin levels above the BALF control and are given as means ± SE of 8 experiments. Linear regression analysis with a 95% confidence interval reveals that, when dose is normalized to surface area of particles administered, responses to UFTiO_2 _and FTiO_2 _are not significantly differently.

Albumin levels in BALF were analyzed to assess air/blood barrier injury after TiO_2 _exposures. Both UFTiO_2 _and FTiO_2 _caused a dose dependent increase in albumin levels. For albumin levels, on a mass dose basis, a significantly greater mass dose of FTiO_2 _was required at all post-exposure time points, to obtain the same responses as seen with UFTiO_2 _exposure (Figure 6). In fact, for albumin levels, UFTiO_2 _exposure was shown to be approximately 82 times more potent than FTiO_2 _at 1 day post-exposure and 41 times more potent at 7 and 42 days post-exposure when mass was the dose metric (Table 1). However, when dose of particles was normalized to surface area of particles administered the difference in albumin levels of the two particle types was much less (Figure 7). Indeed, a linear regression curve analysis with a 95% confidence interval showed that there was no significant difference between the dose-response curves for UFTiO_2 _and FTiO_2 _when dose was expressed as total particle surface area delivered (Figure 7). When dose was normalized to surface area of particles administered, the UFTiO_2 _exposure produced albumin levels that were at most only 3 fold greater than the FTiO_2 _exposure at all post-exposure time points analyzed (Table 1).

IL-1β, TNF-α, and MIP-2 levels were also measured in response to various mass doses of UFTiO_2 _and FTiO_2 _at all post-exposure time points (data similar to Figures 2, 4 and 6 not shown). The highest dose of UFTiO_2 _caused a significant increase in TNF-α, IL-1β and MIP-2 mediator levels over control values at all post-exposure time points (Table 2). The high dose of FTiO_2 _caused a significant increase in MIP-2 and TNF-α levels at all post-exposure time points and caused a significant increase in IL-1β cytokine levels at 7 days and 42 days post-exposure (Table 2). For all three mediators, a significantly greater mass dose of FTiO_2 _was required to obtain the comparable levels in the BAL fluid as elicited by UFTiO_2 _exposure (Table 1). For TNF-α cytokine levels, on a mass dose basis, UFTiO_2 _was shown to be 41 times more potent than FTiO_2 _at all post-exposure time points (Table 1). However, when dose was normalized to surface area of particles administered, TNF-α cytokine levels produced by UFTiO_2 _exposure were at most only 1.5-fold greater than FTiO_2 _levels. For MIP-2 levels, on a mass dose basis, UFTiO_2 _was shown to be 41 times more potent than FTiO_2 _at 1 and 7 days post-exposure and 82 times more potent at 42 days post-exposure (Table 1). However, when dose was normalized to surface area of particles administered, MIP-2 levels produced by UFTiO_2 _exposure were at most only 1.2-fold greater than FTiO_2 _levels (Table 1). In regards to IL-1β cytokine levels, on a mass dose basis, UFTiO_2 _was shown to be 82 times more potent than FTiO_2 _at all post-exposure time points (Table 1). However, when dose was normalized to surface area of particles administered, IL-1β cytokine levels produced by UFTiO_2 _exposure were at most only 1.8-fold greater than FTiO_2 _levels (Table 1). Dose response curves assessing TNF-α, IL-1β, and MIP-2 levels based on surface area of particles administered between UFTiO_2 _and FTiO_2 _exposure were assessed using a linear regression curve analysis with a 95% confidence interval (data similar to Figures 3, 5 and 7 not shown). This analysis showed there was no significant difference in the potency of UFTiO_2 _vs FTiO_2 _for any of the mediators between the two curves at any post-exposure time point.

**Table 2:** Effect of Exposure to UFTiO_2 _vs. FTiO_2 _(High Dose) on Pulmonary Responses.

**Parameter**	**Group**	**1 day**	**7 days**	**42 days**
**PMN (10^6^)**	**Control**	1.4 ± 0.1	0.78 ± 0.07	0.88 ± 0.10
	**UFTiO2 high**	23.8 ± 1*	25.5 ± 3*	18.3 ± 1*
	**FTiO_2 _high**	15.6 ± 0.9*	19.8 ± 0.7*	10.8 ± 0.4*
				
**LDH (U/l)**	**Control**	46.3 ± 2	39.5 ± 1.35	37.3 ± 2
	**UFTiO2 high**	337.4 ± 3*	323.5 ± 3*	239.9 ± 5*
	**FTiO_2 _high**	307.6 ± 5*	288.1 ± 5*	175.6 ± 4*
				
**Albumin**	**Control**	0.07 ± 0.03	0.08 ± 0.003	0.09 ± 0.007
	**UFTiO2 high**	0.38 ± 0.04*	0.51 ± 0.02*	0.40 ± 0.01*
	**FTiO_2 _high**	0.21 ± 0.055*	0.40 ± 0.01*	0.30 ± 0.01*
				
**TNF-α (pg/ml)**	**Control**	25.7 ± 0.6	23.1 ± 0.7	25.86 ± 0.8
	**UFTiO2 high**	67.4 ± 2.2*	73.5 ± 1.5*	88.5 ± 4.4*
	**FTiO_2 _high**	60.7 ± 1.7*	58.5 ± 2.8*	55.4 ± 1.3*
				
**MIP-2 (pg/ml)**	**Control**	517.9 ± 12	601.8 ± 26	643.6 ± 7
	**UFTiO2 high**	802.9 ± 28*	886.9 ± 21*	893.5 ± 26*
	**FTiO_2 _high**	695.6 ± 15*	847.1 ± 43*	852.1 ± 22*
				
**IL-1β (pg/ml)**	**Control**	75.9 ± 6	82.2 ± 6	91.9 ± 8
	**UFTiO2 high**	167.5 ± 13*	230.4 ± 14*	273.6 ± 14*
	**FTiO_2 _high**	119.9 ± 13	131.5 ± 11*	180.3 ± 14*
				
**Zym. Stim. Chemi.**	**Control**	0.53 ± .08	0.50 ± .05	0.51 ± .05
	**UFTiO2 high**	4.4 ± .51*	4.4 ± .47*	4.2 ± .48*
	**FTiO_2 _high**	2.6 ± .26*	2.8 ± .35*	1.4 ± .27
				
**NO Dep. Chemi.**	**Control**	4.3 ± .06	0.45 ± .11	0.39 ± .08
	**UFTiO2 high**	3.3 ± .11*	2.2 ± .27*	1.0 ± .12*
	**FTiO_2 _high**	2.6 ± .20*	0.28 ± .04	0.230 ± .04

Comparison of pulmonary toxicity parameters measured in control animals and for animals receiving the high doses (1.04 and 21.41 mg/rat) of UFTiO_2 _and FTiO_2 _at all post-exposure time points. * denotes that the change from control is statistically significant (p < 0.05) using ANOVA.

Alveolar macrophage zymosan-stimulated and NO-dependent chemiluminescence were measured to assess reactive oxygen species production after UFTiO_2 _and FTiO_2 _exposures. On a mass dose basis, UFTiO_2 _and FTiO_2 _both caused dose-dependent increases in zymosan-stimulated and NO dependent chemiluminescence (data analogous to Figures 2, 4 and 6 not shown). The responses to the high dose are shown in Table 2. A significantly greater mass dose of FTiO_2 _was required to produce comparable chemiluminescence levels as elicited by UFTiO_2 _exposure. The highest doses of UFTiO_2 _caused a significant increase in zymosan and NO-dependent chemiluminescence levels over control at all post-exposure time points (Table 2). The high dose of FTiO_2 _caused a significant increase over control in zymosan-stimulated chemiluminescence at 1 and 7 days post-exposure and in NO-dependent chemiluminecence only at 1 day post-exposure (Table 2).

On a mass dose basis, in regards to zymosan-stimulated chemiluminescence levels, UFTiO_2 _was shown to be 82 times more potent than FTiO_2 _at all post-exposure time points (Table 1). However, when dose was normalized to surface area of particles administered, UFTiO_2 _zymosan-stimulated chemiluminecence levels were at most only 3 fold higher than FTiO_2 _zymosan-stimulated chemiluminecence levels (Table 1). When the surface area zymosan-stimulated chemiluminescence dose response curves were analyzed using a linear regression curve with a 95% confidence interval (data not shown), there was no significant difference between the UFTiO_2 _and FTiO_2 _dose response curves at 1 day or 7 days post-exposure.

On a mass dose basis, in regards to NO-dependent chemiluminescence levels, UFTiO_2 _was 41 times more potent than FTiO_2 _at 1 day post-exposure and was 82 times more potent at 7 and 42 days post-exposure. However, when dose was normalized to surface area of particles administered, UFTiO_2 _NO-dependent chemiluminescence levels were only 1.5-fold greater than FTiO_2 _at 1 day post-exposure but were 8-fold greater at 42 days post-exposure (Table 1). When the surface area NO-dependent chemiluminescence dose response curves were analyzed using a linear regression curve with a 95% confidence interval (data not shown), there was no significant difference between the UFTiO_2 _and FTiO_2 _dose response curves at 1 day post-exposure.

Pulmonary fibrosis

At 42 days post-exposure, lungs were evaluated for pulmonary fibrosis by histological examination of lung tissue stained with Masson's trichrome. No excess fibrotic tissue was noted in lungs exposed to either UFTiO_2 _or FTiO_2 _(micrographs not shown).

### Comparison of lung burden data from TiO_2 _metal analysis

Rats were exposed to the medium dose of UFTiO_2 _or FTiO_2 _(0.52 mg/rat or 10.7 mg/rat, respectively). This mass exposure resulted in the same particle surface area delivered to the lung (0.06 cm^2 ^particles/cm^2 ^alveolar epithelial surface). The amount of UFTiO_2 _or FTiO_2 _in the lung was determined at 7 day and 42 day post-exposure. The metal analysis results indicate that the amount of UFTiO_2 _remaining in the lung decreased by 51% from the 7 day to the 42 day post-exposure time period, while the lung burden of FTiO_2 _decreased by 12% (Table 3).

**Table 3:** Decline in lung burden from 7 to 42 days post – exposure

**Particle**	**Percent decline**
UFTiO_2_	51 ± 4%

FTiO_2_	12 ± 1%

Values are for medium dose exposures (0.52 mg/rat vs 10.7 mg/rat for UFTiO_2 _and FTiO_2_, respectively, i.e., a surface area dose of 0.06 cm^2 ^particles/cm^2 ^alveolar epithelial surface). Values are means ± SE of at least 4 experiments.

### Comparison of lymph node burden data from TiO_2 _metal analysis

To determine if the decline in lung burden was due to migration of particles to the lymph nodes, the amount of TiO_2 _present in the tracheo-bronchial (TBL) and thymic lymph nodes (TLN) was assessed at days 7 and 42 post-exposure. The results indicate that lymph node burden for UFTiO_2 _from 7 to 42 days post-exposure increased by 246%, while a 134% increase was observed for FTiO_2 _(Table 4). Therefore, although relative migration of UFTiO_2 _to the lymph nodes exceeded that for FTiO_2_, this difference could not account for the much lower lung clearance of FTiO_2 _relative to UFTiO_2 _(Table 3).

**Table 4:** Increase in lymph node burden from 7 to 42 days post-exposure

**Particle**	**Percent increase**
UFTiO_2_	246 ± 118%

FTiO_2_	134 ± 2%

Values are for medium dose exposures (0.52 mg/rat vs 10.7 mg/rat for UFTiO_2 _and FTiO_2_, respectively, i.e., a surface area dose of 0.06 cm^2 ^particles/cm^2 ^alveolar epithelial surface). Values are means ± SE of at least 4 experiments.

### Comparison of lavaged lung burden data from TiO_2 _metal analysis

Lung burden of TiO_2 _was measured in both lavaged and unlavaged lungs at 7 and 42 days post-exposure. The amount of TiO_2 _present in the lavaged lung is the non-lavagable fraction, i.e., the fraction not phagocytized by lavagable alveolar macrophages or present as free particles in the airspaces. The non-lavagable fraction would be expected to increase if particles migrated to the interstitium. The non-lavagable UFTiO_2 _or FTiO_2 _present in the lavaged lung increased from 7 to 42 days post-exposure (Table 5). However, there was a striking difference between the lavagable vs non-lavagable component of UFTiO_2 _compared to FTiO_2_. With UFTiO_2_, the majority of the particles were non-lavagable, suggesting migration to the interstitium. In contrast, the majority of FTiO_2 _was found in lavagable alveolar macrophages at both 7 and 42 days post-exposure.

**Table 5:** Percent lavagable vs non-lavagable TiO2 in the lung

**Particle**	**7 Days post**	**42 Days post**
UFTiO_2_	19% lavagable	0% lavagable
	81% non-lavagable	100% non-lavagable

FTiO_2_	91% lavagable	84% lavagable
	9% non-lavagable	16% non- lavagable

Values are for medium dose exposures (0.52 mg/rat vs 10.7 mg/rat for UFTiO_2 _and FTiO_2_, respectively, i.e., a surface area dose of 0.06 cm^2 ^particles/cm^2 ^alveolar epithelial surface).

## Discussion

Fine TiO_2 _particles as well as other low-solubility, low-toxicity particles have long been of interest in particle toxicology. Historically, fine TiO_2 _particles were utilized as negative controls in numerous pulmonary toxicity studies. However, the current study shows that FTiO_2 _at relatively high mass lung burdens can induce pulmonary inflammation and damage, which are sustained through 42 days post-exposure. Using a mass dose metric, UFTiO_2 _was significantly more potent (at least 41 fold) than FTiO_2 _in causing sustained lung damage and inflammation. When exposure dose was normalized to equivalent surface area of particles delivered, no significant difference in potency between UFTiO_2 _and FTiO_2 _was observed. These findings help support the hypothesis that surface area of particles administered may be the more appropriate dose metric for nanoparticle pulmonary toxicity studies. This conclusion, although not novel, has been recently disputed [12,13].

Two similar studies were conducted by Warheit et al. [12,13] to assess the lung toxicity of intratracheally instilled nano-sized and fine-sized TiO_2_. In both studies, they instilled fine and ultrafine TiO_2 _suspended in PBS into rats (1 or 5 mg/kg) and compared measured pulmonary parameters to animals instilled with crystalline silica (used as a positive control). They monitored inflammation as PMN influx and cytotoxicity as LDH activity at 1 day, 1 week, 1 month and 3 months post-exposure in both studies. Warheit et al. [12,13] concluded that exposures to nanosized TiO_2 _had no significant long term adverse pulmonary effects. They also reported similar acute levels of inflammation and cytotoxicity for both nano-sized and fine-sized TiO_2_, with only the highest dose (5 mg/kg) at 1 day post-exposure, causing a significant increase in inflammation for both nano- and fine-sized TiO_2 _exposures. Warheit and colleagues argued that the transient, reversible inflammation seen at 1 day post-exposure with both nano-sized and fine-sized TiO_2 _was due primarily to the method of exposure (intratracheal instillation) rather than the effects of the nano-sized particles in the lung. The inflammation shown at 1 day post-exposure in the Warheit et al. [12,13] studies quickly resolved to control levels by 1 week post-exposure.

In the first study, Warheit et al. [12] concluded that their results "run counter to the postulation that surface area is a major factor associated with the pulmonary toxicity of nanoscale particle-types". They argued that their study was instrumental in demonstrating that the fact that nanoparticles have larger surface areas does not necessarily indicate that they produce increased pulmonary inflammation and cytotoxicity when compared to fine-sized particles of similar composition. Warheit et al. [13], using very similar data to the Warheit et al. [12] study, expanded on the conclusions from the initial study. They again concluded that the fact that nanoparticles have larger surface areas does not necessarily indicate that they produce increased pulmonary inflammation and cytotoxicity when compared to fine-sized particles of similar composition.

The findings of the current study disagree with the findings of Warheit et al. [12,13]. The results of the current study indicate that surface area of the nanoparticles does in fact play a role in pulmonary toxicity, and that low mass burdens (as low as 0.26 mg/rat) of UFTiO_2 _can cause persistent lung damage and inflammation over a 42 day post-exposure study period. The results of the current study support the fact that on a mass dose basis nano-sized particles are more toxic than fine-sized particle of the same composition. However, when dose is normalized to surface area of particles administered, the difference in toxicity responses between UFTiO_2 _and FTiO_2 _for all pulmonary parameters measured becomes substantially less. This, therefore, indicates that surface area of particles is a critical driver of toxicity and may be an appropriate dose metric for pulmonary toxicity studies. The discrepancies between the two studies are believed to be due to the fact that Warheit et al. [12,13] reports extreme agglomeration of the PBS-suspended nano-sized TiO_2 _utilized in their studies. In addition, significant agglomeration was also reported for the suspension of fine TiO_2_. This agglomeration issue leads to an over-estimation of delivered surface area of the particles. Indeed, their dynamic light scattering data indicate that once suspended in PBS both ultrafine and fine TiO_2 _had agglomerated particle sizes of > 2 μm. One would argue that since delivered particle size was equivalent, toxicity would not be expected to differ. In the present study, efforts were made to substantially improve the dispersion of UFTiO_2 _and FTIO_2 _by using rat bronchoalveolar lavage fluid (BALF) as the suspension medium. Results from our laboratory indicate that dispersion was vastly improved for both UFTiO_2 _and FTiO_2 _in BALF compared to PBS [14]. Our laboratory has reported that the diameter determined by dynamic light scattering of UFTiO_2 _suspended in a BALF model medium was 204 nm [16]. Therefore, in the present study, a potency difference between UFTiO_2 _and FTiO_2 _(on a mass basis) was observed because the physical surface areas of the UF vs F structures were different.

A recent study by Duffin et al. [17] further supports the conclusions of the current study. They investigated the relationship between inflammation, as indicated by neutrophil content of the bronchoalveolar lavage of rats 18–24 hours after particle instillation, and particle size. Rats were instilled with ultrafine and fine-sized carbon black or titanium dioxide and then assessed for PMN influx into the lung. Duffin et al. [17] reported that the extent of inflammation was a function not of mass dose instilled but of the surface area of particles instilled. Likewise, the findings of the current study support the conclusion that inflammatory potential of TiO_2 _is a function of surface area of the particles administered.

Secondly, studies by Warheit et al. [12,13] report that UFTiO_2 _at lung burdens of 1–5 mg/kg did not cause persistent lung damage and inflammation. In contrast, data from the present study indicate that lung burdens as low as 1 mg/kg UFTiO_2 _caused significant lung damage and inflammation which persisted through 42 days post-exposure. In the present study, efforts were made to improve dispersion of UFTiO_2 _by using BALF as the suspension medium, as reported previously [14]. Shvedova et al. [18] reported that improved dispersion of ultrafine carbon black (UFCB) particles in BALF increased the inflammatory and damage potency compared to UFCB suspended in PBS; i.e., intratracheal instillation of a 30 fold greater mass of poorly dispersed UFCB suspended in PBS was required to attain the same level of pulmonary damage (LDH activity) and inflammation (PMN level) as well dispersed UFCB suspended in BALF in a rat model. Therefore, poor dispersion of UFTiO_2 _in PBS (diameters of over 2 μm determined by dynamic light scattering) may account for the low biological activity previously reported by Warheit et al. [12,13].

Warheit et al. [13] suggested that particle characteristics, such as surface reactivity, crystal structure, surface pH, surface coating, shape, etc., may play important roles in the pulmonary toxicity of nanoparticles. They have demonstrated that UFTiO_2 _in the uncoated, 80/20 anatase/rutile form exhibited greater surface activity than alumina-coated rutile as judged by ability to deplete vitamin C in an acellular assay. In addition, Sayes et al. [19] demonstrated that 80/20 anatase/rutile generated 6 fold more reactive species (measured as acellular luminol-enhanced chemiluminescence) than rutile after UV irradiation. They argued that this increased surface reactivity resulted in greater *in vitro* cytotoxicity for the anatase form of UFTiO_2. _In the present study, the UFTiO_2 _used was 80/20 anatase/rutile, while the FTiO_2 _was the rutile crystal form. However, the particle samples were not irradiated with UV light in this study. In the absence of UV irradiation, Sayes et al. [19] reported the UFTiO_2 _in the 80/20 anatase/rutile form generated only twice the reactive species as ultrafine rutile. Such as small difference in surface reactivity can not account for the 40 fold greater potency of ultrafine vs. fine TiO_2 _on an equal mass basis reported in the present study. However, a 2 fold difference in surface activity may explain why normalizing dose to equivalent particle surface area delivered did not result in identical potency for the ultrafine and fine particle samples. Recent results indicate that the shape of nanoparticles can affect toxic potential. Porter et al. [20] demonstrated that TiO_2 _nanowires exhibited greater toxicity *in vitro* and greater inflammatory potential *in vivo* than TiO_2 _nanospheres of the same crystal structure and diameter. Therefore, although the present study concentrated on the role of particle surface area in nanoparticle toxicity, results do not discount the importance of other particle characteristics in nanoparticle toxicity.

Results of lung burden studies suggest that although a large portion of FTiO_2 _appears to have been engulfed by lavagable alveolar macrophages (Table 5) clearance of FTiO_2 _was relatively low, 12% from 7 to 42 days post-exposure (Table 3). One, thus, could conclude that the lungs were in overload at this FTiO_2 _burden (10.7 mg/rat). As proposed by Morrow [10], when alveolar macrophages have engulfed a large volume of particles, the phagocytes become immobile and clearance fails. In contrast, macrophage clearance apparently is relatively robust after exposure to 0.52 mg/rat UFTiO_2_; i.e., 51% decline in lung burden is observed from 7 to 42 days post-exposure (Table 3). This clearance is only partially due to migration of UFTiO_2 _to the lymph nodes (Table 4). In addition, migration of UFTiO_2 _into the alveolar walls is far more substantial than that for FTiO_2 _(Table 5). A high rate of interstitilization of UFTiO_2 _vs FTiO_2 _has been reported previously [21].

## Conclusion

In summary, exposure of rats by intracheal instillation to a well-dispersed suspension of UFTiO_2 _caused dose dependent pulmonary damage and inflammation, which persisted 42 days post-exposure. On a mass dose basis, UFTiO_2 _was at least 41 fold more potent than FTiO_2_. When exposure dose was normalized to surface area of particles delivered, this potency difference was no longer significant. Burden data indicate that UFTiO_2 _migrates to the interstitium to a far greater extent than FTiO_2_. Results support the use of particle surface area as a dose metric for evaluation of pulmonary response to particles.

## Methods

### Experimental design

Recently, the hypothesis that nanoparticles exhibit unique bioactivity due to their large surface area per mass has been questioned [12,13]. The aim of the present study was to readdress the issue of the relative toxicity of UFTiO_2 _vs FTiO_2 _on a mass dose or equivalent particle surface area delivered dose basis. In this study, particle dispersion was substantially improved by suspension of particles in diluted alveolar lining fluid obtained by bronchoalveolar lavage of naïve rats as described previously [14]. Rats were exposed to various mass concentrations of UFTiO_2 _vs FTiO_2 _by intratracheal instillation. Mass doses of UFTiO_2 _(0.26, 0.52 and 1.04 mg/rat) and FTiO_2 _(5.35, 10.7 and 21.41 mg/rat) were chosen to result in equivalent total particle surface area delivered doses (0.0313, 0.0625 and 0.125 cm^2 ^of particles administered per cm^2 ^of alveolar epithelial surface) for UFTiO_2 _and FTiO_2_. Rats were sacrificed at 1 day, 7 days, and 42 days post-exposure, and markers of inflammation (PMN number or inflammatory mediator levels in BAL samples), lung injury (LDH activity or albumin levels in BAL samples), and macrophage activity (zymosan-stimulated or NO-dependent chemiluminescence) were monitored. The magnitude of pulmonary responses to exposure to UFTiO_2 _vs FTiO_2 _was then compared on a mass dose and an equivalent surface area of particles administered dose basis to evaluate the role of particle surface area in the pulmonary response.

### Animals for *in vivo* exposures

The rats used for the *in vivo* experiments were male Fischer CDF (F344/DuCrl) rats weighing 200–300 g (~10 weeks old at arrival) obtained from Charles River (Raleigh, NC). The animals were housed in an AAALAC-accredited; specific pathogen-free, environmentally controlled facility. The animals were monitored to be free of endogenous viral pathogens, parasites, mycoplasms, Helicobacter and CAR Bacillus. Animals were housed in ventilated cages which were provided HEPA-filtered air, with Alpha-Dri virgin cellulose chips and hardwood Beta-chips used as bedding. The rats were maintained on a Teklad 2918 diet and tap water, both of which were provided ad libitum.

### Bronchoalveolar fluid collection for particle suspension media

Rats were euthanized with an i.p. injection of sodium pentobarbital (>100 mg/kg body weight) and exsanguinated by cutting the descending aorta. A tracheal cannula was inserted and bronchoalveolar lavage was conducted [22]. A 6 ml aliquot of cold Ca^+2 ^and Mg^+2^-free phosphate-buffered saline (PBS) was used for the lavage wash. The cold PBS was flushed into and out of the lungs two times before the lavage fluid was collected. The bronchoalveolar lavage (BAL) from five rats was combined and centrifuged at 600 × g for 10 minutes using a Sorvall RC 3B Plus centrifuge (Sorvall Thermo Electron Corporation, Asheville NC). The supernatant was decanted into a new tube, while the pellet was discarded. This acellular bronchoalveolar lavage fluid (BALF) was then used as the vehicle for particle suspensions. The BALF was collected fresh the same day that the particulate suspensions were made.

### Particles

UFTiO_2 _(Aeroxide TiO_2 _P-25) was obtained as a gift from Degussa Corporation (Parsippany, NJ). UFTiO_2 _had a primary particle size of 21 nm and was a 80/20 mixture of anatase/rutile. FTiO_2 _(titanium (IV) oxide, #224227) was purchased from Sigma-Aldrich (St. Louis, MO). FTiO_2_had a primary particle size of 1 μm and was 100% rutile.

### Suspension of UFTiO_2 _and FTiO_2 _in rat BALF

Ultrafine titanium dioxide (UFTiO_2_) and fine titanium dioxide (FTiO_2_) particles were sieved using a Retsch AS 200 Sieve (Retsch GmbH, Haann, Germany) through 1.18 mm, 250 μm, and 45 μm mesh screens. Particles were weighed and suspended in rat BALF to obtain the desired concentration. Once the particles were added to the BALF, the suspensions were pulse sonicated with 5 individual pulses at a duty cycle setting of 10% and an output setting of 1 with a Branson 450 Sonifier probe sonicator (Branson Ultrasonics Corporation, Danbury CT.). This method has been reported previously by our laboratory to result in well dispersed suspensions as determined by light and electron microscopy [14]. Dynamic light scattering of UFTiO_2 _suspended in a BALF model medium indicated that ultrafine particles had a diameter of 204 ± 18 nm [16].

### *In vivo* exposures

To receive their respective dose of particles, each rat was anesthetized with an intraperitoneal (i.p.) injection of methohexital sodium (30–40 mg/kg body weight; Monarch Pharmaceuticals, Bristol, TN). Each animal was then instilled via intratracheal instillation (IT) using a 20-gauge 4-inch ball tipped animal feeding needle [22]. Each animal was instilled with 0.3 ml of UFTiO_2 _or FTiO_2 _suspended in BALF. BALF (0.3 ml) was instilled as the control. Results from our laboratory indicate that instillation of BALF does not cause lung inflammation [14]. In addition, suspension of quartz in BALF did not alter its inflammatory potency [14].

### Bronchoalveolar lavage and cell differentials

At 24-hours, 7 days, and 42 days post-IT, the animals were euthanized with an i.p. injection of sodium pentobarbital (>100 mg/kg body weight) and exsanguinated by cutting the descending aorta. A tracheal cannula was inserted and bronchoalveolar lavage (BAL) was conducted [22]. A 6 ml aliquot of cold Ca^+2 ^and Mg^+2 ^free PBS was used for the first lavage wash. The cold PBS was flushed into and out of the lungs two times before the lavage fluid was collected. After the first lavage wash was collected and stored separately, the BAL continued with 8 ml aliquots of cold Ca^+2 ^and Mg^+2^-free PBS until an additional 80 ml of bronchoalveolar lavage was collected. The BAL was then centrifuged at 600 × g for 10 minutes using a Sorvall RC 3B Plus centrifuge (Sorvall Thermo Electron Corporation, Asheville NC). After centrifugation, the supernatant from the first lavage wash was decanted into a clean conical vial and was stored on ice to be used for lung damage/cytotoxicity analysis. The remaining supernate from the lavages was discarded, and the cells remaining were washed with cold Ca^+2 ^and Mg^+2^-free PBS and spun again at 600 × g for 10 minutes. After this, the supernatant was discarded and the cells were resuspended in 1 ml of HEPES-buffered medium. Using these lavage samples, polymorphonuclear neutrophil (PMN) and alveolar macrophage (AM) counts were determined to assess inflammation. The number of AM and PMN was determined according to their unique cell diameters, using an electronic cell counter equipped with a cell sizing attachment (Beckman Coulter Multisizer 3 Counter, Hialeah, FL).

### BAL fluid lactate dehydrogenase activity and albumin concentration

The degree of cytotoxicity induced by the instilled particles was determined by lactate dehydrogenase (LDH) activity in the BALF. LDH activity was measured using a Roche COBAS MIRA Plus chemical analyzer (Roche Diagnostic Systems Inc., Branchburg, NJ) as described previously by our laboratory [22]. Albumin concentrations in BALF were assessed to examine if instilled particle exposures had compromised the integrity of the alveolar air/blood barrier. Albumin concentrations were also measured using a Cobas Fara II Analyzer (Roche Diagonostic Systems, Montclair, NJ) as previously described by our laboratory [22].

### Mediator measurements in bronchoalveolar lavage fluid

The presence of inflammatory mediators in the BALF was analyzed by enzyme-linked immunosorbent assay (ELISA). The levels of mediators present were measured using commercially available ELISA kits (BioSource International Inc., Camarillo, CA). Three mediators were quantified: tumor necrosis factor-α (TNF-α), interleukin (IL)-β, and macrophage-inflammatory protein-2 (MIP-2).

### Zymosan-stimulated and NO-dependent alveolar macrophage chemiluminescence

Reactive oxygen species production was determined by measuring AM chemiluminescence. According to Van Dyke et al. [23], only AM will generate reactive oxygen species in response to unopsonized zymosan in the chemiluminecence assay procedure. The AM chemiluminescence assay was conducted in the same manner as previously described by our laboratory [22]. Briefly, resting AM chemiluminescence was determined by incubating 1.0 × 10^6 ^AM/ml at 37°C for 20 minutes, followed by the addition of 5-amino-2,3-dihydro-1,4, phthalazinedione (luminol) to a final concentration of 0.08 μg/ml. This was then followed by the measurement of chemiluminescence generated over 15 minutes.

Zymosan-stimulated chemiluminescence was determined by adding unopsonized zymosan (2 mg/ml) to the AM samples immediately prior to measurement of chemiluminescence. NO-dependent chemiluminescence was determined by adding the unopsonized zymosan as well as N-nitro-L-arginine methyl ester HCL (L-NAME) to the AM samples immediately prior to measurement of chemiluminescence. Zymosan-stimulated (zymosan – rest) and NO-dependent (zymosan – zymosan with L-NAME) chemiluminescence were both measured using a Berthold automated luminometer (Berthold Autolumat LB 953, EG&G, Gaithersburg, MD) at 390–620 nm for 15 minutes.

### Tissue collection and analysis for titanium dioxide burden analysis

Immediately after the 7 and 42- day exposure time periods, rats were euthanized with an i.p. injection of sodium pentobarbital (>100 mg/kg body weight). The left and right lungs as well as the tracheobronchial (TBL) and thymic (TLN) lymph nodes were removed for titanium dioxide burden analysis. After removal, the lungs and lymph nodes were weighed and then frozen at -80°C. The lungs and lymph nodes were then lyophilized. The lyophilized samples were sent to Bureau Veritas North America, Inc. (Novi, MI) for TiO_2 _metal analysis.

### Histopathology

Immediately after the 7 and 42 days exposure, rats were euthanized with i.p. injection of sodium pentobarbital (> 100 mg/kg body weight). The lungs were removed and infused to 25 cm H_2_O with 10% buffered neutral formalin. Paraffin-embedded histologic sections were stained with hematoxylin and eosin (H&E) or Masson's trichrome for light microscopic examination by a board-certified veterinary pathologist.

### Statistics

Statistical differences between control groups and treatment groups for the *in vivo* experiments examining the toxicity of titanium dioxide were determined using an analysis of variance (ANOVA). Individual means were compared using the Student-Newman-Keuls Method multiple comparison procedure with an overall significance level of p= 0.05. A linear regression curve analysis with a 95% confidence interval was also conducted on the surface area data for each pulmonary parameter measured.

#### Disclaimer

The findings and conclusions in this report are those of the authors and do not necessarily represent the views of the National Institute for Occupational Safety and Health.

## Abbreviations

AAALAC: Association for Assessment and Accreditation of Laboratory Animal Care; AM: Alveolar macrophage; ANOVA: Analysis of variance; BAL: Bronchoalveolar lavage; BALF: Bronchoalveolar lavage fluid; BET: Brunauer-Emmett-Teller; Ca^+2^: Calcium; CB: Carbon black; cm: centimeter; CVF: Certified virus free; FCB: Fine carbon black; FTiO2: Fine titanium dioxide; H&E: Hematoxylin and eosin; IL: Interleukin; i.p.: intraperitoneal; IT: Intratreacheal instillation; LDH: Lactate dehydrogenase; Mg^+2^: Magnesium; μm: Micrometer; mg: Milligram; MIP: Macrophage inflammatory protein; ml: Milliliter; SE: Standard error; TBL: Tracheo-bronchial lymph node; TiO_2_: Titanium dioxide; TLN: Thymic lymph node; TNF: Tumor necrosis factor; UF: Ultrafine; UFCB: Ultrafine carbon black; UFTiO_2_: Ultrafine titanium dioxide.

## Competing interests

The authors declare that they have no competing interests.

## Authors' contributions

TMS carried out all of the *in vivo* experiments involved in this study including the intratracheal instillations and animal sacrifices. TMS drafted the manuscript and performed the statistical analysis. CK conducted the histopathology analysis for the study. Both TMS and VC conceived of the study and participated in its design. VC participated in the study coordination, data analysis and interpretation, and helped draft the manuscript. All authors read and approved the final manuscript.
